# Down-regulation of cyclin D2 in amyloid β toxicity, inflammation, and Alzheimer’s disease

**DOI:** 10.1371/journal.pone.0259740

**Published:** 2021-11-18

**Authors:** Grzegorz A. Czapski, Magdalena Cieślik, Emilia Białopiotrowicz, Walter J. Lukiw, Joanna B. Strosznajder

**Affiliations:** 1 Department of Cellular Signalling, Mossakowski Medical Research Institute, Polish Academy of Sciences, Warsaw, Poland; 2 Laboratory of Neurodegeneration, International Institute of Molecular and Cell Biology, Warsaw, Poland; 3 LSU Neuroscience Center and Departments of Neurology and Ophthalmology, Louisiana State University School of Medicine, New Orleans, LA, United States of America; Nathan S Kline Institute, UNITED STATES

## Abstract

In the current study, we analyzed the effects of the systemic inflammatory response (SIR) and amyloid β (Aβ) peptide on the expression of genes encoding cyclins and cyclin-dependent kinase (Cdk) in: **(i)** PC12 cells overexpressing human beta amyloid precursor protein (βAPP), wild-type (APPwt-PC12), or carrying the Swedish mutantion (APPsw-PC12); **(ii)** the murine hippocampus during SIR; and **(iii)** Alzheimer’s disease (AD) brain. In APPwt-PC12 expression of cyclin D2 (cD2) was exclusively reduced, and in APPsw-PC12 cyclins cD2 and also cA1 were down-regulated, but cA2, cB1, cB2, and cE1 were up-regulated. In the SIR cD2, cB2, cE1 were found to be significantly down-regulated and cD3, Cdk5, and Cdk7 were significantly up-regulated. Cyclin cD2 was also found to be down-regulated in AD neocortex and hippocampus. Our novel data indicate that Aβ peptide and inflammation both significantly decreased the expression of cD2, suggesting that Aβ peptides may also contribute to downregulation of cD2 in AD brain.

## Introduction

Alzheimer’s disease (AD) is the most common age-related neurodegenerative disorder leading inevitably to progressive and severe dementia and death. Multiple processes, pathways and systems have been shown to be impaired in AD, but despite many years of extensive research the etiology and pathomechanism of AD remain elusive, and effective remedial treatments have not been established. Neuropathologically, AD brains are characterized by the presence of extracellular amyloid plaques and intracellular neurofibrillary tangles (NFT) which are composed of amyloid β (Aβ) peptides and hyperphosphorylated MAP tau protein, respectively. Aβ peptides are derived from the beta amyloid precursor protein (βAPP) after cleavage by γ- and β- secretases. In the early-onset (genetic) form of AD, mutations in genes coding βAPP or presenilins are responsible for the overproduction of Aβ peptides. In the late-onset (sporadic) form of AD the trigger(s) for overproduction of Aβ remain unknown [1]. Several recent discoveries have highlighted the role of inflammatory processes in the initiation or propagation of AD-related pathology. The data suggest that depending on the disease stage and a patient’s health status, activation of the immune system may be protective or detrimental, or even may significantly contribute to the triggering of AD pathology [2–7].

A growing body of evidence indicates that re-activation of cell cycle in mature post-mitotic neurons may be a critical pathogenetic event in AD [8–11]. Terminally differentiated neurons exist in a quiescent state of the cell cycle; however, several studies have demonstrated that cell cycle-related proteins are expressed in post-mitotic neurons in healthy adult brain [12]. Because the expression of cell-cycle regulators in healthy differentiated neurons is not related to neuronal proliferation, it was suggested that they may have a non-canonical role, possibly linked to neuronal plasticity or stability. However, a low fraction of aneuploid neurons exist in the healthy adult brain. This low-frequency aneuploidy seems to be well tolerated in the brain [11]. However, a loss of cell-cycle control machinery in neurons leads to reactivation of the cell cycle, DNA replication, and significant increase in the number of aneuploid neurons in AD affected tissues [13]. It was demonstrated that cell cycle-positive neurons are prone to apoptotic cell death under stress conditions [14].

In AD, dysregulation of the cell-cycle machinery was also observed in neuronal progenitor cells. The hippocampal neurogenesis in adults occurs in the subgranular zone (SGZ) of the dentate gyrus (DG). Neural stem cells in the SGZ transform into early and late intermediate neuronal progenitors which proliferate but finally exit the cell-cycle, and form postmitotic immature neurons. Neurogenesis has some regenerative potential, but dysregulation of the cell-cycle may lead to generation of novel, aneuploidic neurons [14].

Several factors have been suggested to affect the cell-cycle in neurons: Aβ peptides, oxidative stress, DNA damage and inflammation [14]. The aim of the present study was to analyze the impact of overproduction of βAPP/Aβ peptides or inflammation on the expression of cell-cycle-related proteins. Our results indicate that cyclin D2 (cD2) is significantly down-regulated by the systemic inflammatory response (SIR), βAPP/Aβ peptide toxicity, and in AD.

## Materials and methods

### Chemicals

Reagents for reverse transcription (High capacity RNA-to-cDNA Master mix) and PCR (Gene expression Master mix and SYBR Green Master mix) were obtained from Applied Biosystems (Applied Biosystems, Foster City, CA, USA). Dulbecco’s Modified Eagle’s Medium (DMEM), Fetal Bovine Serum (FBS), Horse Serum (HS), penicillin, streptomycin, G418, L-glutamine, Deoxyribonuclease I, Tri-reagent, DTT, polyethylenoimine (PEI), DMSO and all other common reagents used in these experiments were obtained from Sigma-Aldrich (St. Louis, MO, USA).

### Cell culture

Rat pheochromocytoma non-differentiated PC12 cells and their derivative clones stably expressing human βAPP were a kind gift from Dr. W.E. Müller (University of Frankfurt, Frankfurt am Main, Germany) [15]. Three PC12 cell lines were used: **(i)** cells transfected with the empty vector (PC12); **(ii)** APPwt PC12 cells transfected with human wild-type APP gene; and **(iii)** APPsw PC12 cells transfected with a human βAPP gene containing the Swedish mutated (K670M/N671L) form of the human APP695 isoform. All cell lines were cultured in DMEM supplemented with 10% heat-inactivated FBS, 5% heat-inactivated HS, 2 mM L-glutamine, 50 U/ml penicillin and 50 μg/ml streptomycin in a 5% CO_2_ atmosphere at 37°C. Stably transfected cells were maintained in cell culture media containing 400 μg/ml G418.

### Animals

Experiments were carried out on C57BL/6 mice (*Mus musculus*), supplied by the Animal House of Mossakowski Medical Research Institute, Polish Academy of Sciences (Warsaw, Poland). To avoid variability related to fluctuating hormone levels in females and variability related to aged animals, experiments were performed on 2–3 month-old (20–25 g) male animals. All animals were maintained under controlled temperature (22°C ± 10%) and humidity (55% ± 10%) conditions on a 12-hour light/dark cycle. All experiments conducted on the animals were approved by the IV Local Ethics Committee for Animal Experimentation in Warsaw (permission 26/2007; 18 July 2007), were carried out in accordance with the EU Directive 2010/63/EU for animal experiments, and complied the ARRIVE guidelines. All efforts were made to minimize animal suffering and to reduce the number of animals used. All manipulations were performed gently and rapidly to reduce animal’s stress. To induce systemic inflammatory response (SIR), LPS (from *E*. *coli* serotype O55:B5; toxicity 1.5×10^7^ EU/mg; Sigma, St. Louis, USA) was dissolved in sterile saline and administered intraperitoneally (i.p.) in a dose of 1 mg/kg. An appropriate volume of the solvent (100 μl) was injected into control animals. Twelve hours and 4 days after administration of LPS the animals were deeply anaesthetized with sodium pentobarbital (60 mg/kg b.w., i.p.) and perfused transcardially with 0.1 M sodium phosphate buffer, pH 7.4 at 4°C to remove blood from the brain. Subsequently brains were collected and the hippocampi were dissected and analyzed. Four animals were used in each experimental group for a total of 16 in the whole experiment: [4×NaCl(12 h), 4×LPS(12 h), 4×NaCl(4 days), 4×LPS(4 days)].

### Microarray analysis of murine hippocampus

Microarray analysis was performed in the Laboratory of Microarray Analysis of Center of Excellence BioExploratorium, Department of Biology Warsaw University and Institute of Biochemistry and Biophysics PAS, Warsaw, Poland as described previously [16]. Briefly, total RNA from the mouse hippocampus was isolated and analyzed by using the Affymetrix Gene Chip Mouse genome 430 Ver 2.0 in a Affymetrix GeneChip 3000 scanner according to the recommendations of the Affymetrix Gene Expression Analysis Technical Manual. Analysis was carried out starting from 2 μg of total RNA. Microarray data were converted to CEL files with the use of Affymetrix Expression Console Software. CEL files were imported to Partek Genomics Suite 6.4 for statistical analysis. The data were normalized with the GC-RMA method and log2 transformed. A three-way ANOVA was performed and genes with an FDR <0.05 were considered statistically significant (see below).

### PCR

Cells were washed twice with ice-cold PBS, scraped from the culture dish and centrifuged briefly (3 min, 1000×g). RNA was isolated from the cell pellet using TRI-reagent according to the manufacturer’s protocol (Sigma-Aldrich, St. Louis, MO, USA). Digestion of DNA contamination was performed using DNase I according to the manufacturer’s protocol (Sigma-Aldrich, St. Louis, MO, USA). RNA quantity and quality was controlled by spectrophotometric analysis and gel electrophoresis. The ratio 260/280 nm of ~2.0 and the ratio 260/230 nm in the range of 2.0–2.2 were considered acceptable for further analysis. Integrity of RNA (lack of degradation) was visually inspected after electrophoresis on agarose gels. Reverse transcription was performed using a High Capacity RNA-to-cDNA Master Mix according to the manufacturer’s protocol (Applied Biosystems, Foster City, CA, USA). Quantitative PCR was performed on an ABI PRISM 7500 apparatus. The PCR cycling program was: 95°C for 10 min, 40 cycles at 95°C for 15 seconds, 60°C for 1 min. As a control the expression of *Actb* was analyzed by using pre-developed TaqMan Gene Expression Assay ACTB-4352340E (Applied Biosystems, Foster City, CA, USA) according to the manufacturer’s instructions. Expression of other genes were analyzed by using SYBR Green Master Mix. The sequence of all primer pairs is shown in S1 Table. The relative levels of mRNA were calculated using the ΔΔCt method.

### Cell cycle analysis

Twenty four hours after passaging, cells (1–2×10^6^) were collected and centrifuged for 5 min in 4°C at 400×g. Cell pellets were washed with 1 ml of PBS (400×g; 4°C; 5 min) and fixed by dropwise addition of 900 μl of cold (-20°C) 70% ethanol. After incubation for 1–2 h in 4°C with constant mixing (on rocking platform) the cells were centrifuged (400×g; 4°C; 10 min), and the pellet was washed with 1 ml of PBS (400×g; 4°C; 10 min), dissolved in staining solution (0.1% sodium citrate, 10 μg/ml RNAse, 50 μg/ml propidium iodide in PBS), and incubated in the dark for 20–30 min at room temperature. Flow cytometric analysis was performed on FACS Canto II (BD). During this analysis 10,000 events were gathered, excluding the doublets and debris.

### Studies in human control and AD neocortex and hippocampus

The study was conducted according to the guidelines of the Declaration of Helsinki. The acquisition, handling, experimental, and analytical procedures involving postmortem human brain tissues were carried out in an ethical manner in strict accordance with the ethics review board policies at brain and tissue donor institutions and at the Louisiana State University (LSU) Health Sciences Center. The ethical use of postmortem human brain tissues and their analyses were also carried out in strict accordance with the Institutional Biosafety Committee and the Institutional Review Board Committee (IBC/IRBC) ethical guidelines IBC#18059 and IRBC#6774 at the LSU Health Sciences Center, New Orleans LA 70112 USA.

The expression levels of cyclin D2 (cD2) was examined in the neocortex and hippocampus of moderate-to-late stage sporadic AD patients versus age- and gender-matched short post-mortem interval (PMI) controls in collaboration with LC Sciences (Houston TX, USA). Briefly, the AD group (n = 5) had a mean age of 75.2±14.5 years and a mean PMI of ~3.3 hours and the control group (N = 4) had a mean age of 74.3±11.8 years and mean PMI of ~3.5 hours; all brain samples were from female donors; there was no significant difference in the mean age, gender, PMI or yield of total RNA between the AD and control groups. Informed consent from next of kin was obtained at brain and tissue donor institutions for all tissue samples prior to autopsy and donation; coded postmortem brain tissue samples (containing no personal identifying information of the donors) were obtained from the brain and tissue banks listed above.

Total RNA was extracted and probed for cyclin D2 and quantified based on unchanging β-actin control levels in the same sample using methods explained in detail in previously published articles from our laboratories [16,17]. Total RNA was quality controlled and exhibited A260/A280 ratios between ~1.9–2.1 indicating highly purified total RNA useable for this type of gene expression analysis. The utilization of DNA, messenger RNA (mRNA) and microRNA arrays and the methodology for total RNA extraction, quality control and gene expression analysis have been extensively described in several of our earlier publications [17–20]. A diagnosis of AD was determined using the clinical dementia rating (CDR) of AD and control patients and/or Alzheimer’s disease diagnostic guidelines/National Institute on Aging criteria (www.nia.nih.gov/health/alzheimers-disease-diagnostic-guidelines). AD dissection, diagnosis and initial tissue characterization was performed and determined at the Alzheimer’s Disease Research Center (ADRC) at the University of California Irvine Institute for Memory Impairments and Neurological Disorders (UCI MIND), a designated Center of Excellence funded by the National Institute on Aging (NIA) at the National Institutes of Health (NIH, Bethesda MD USA). Brain hippocampal and neocortical tissues and/or total nucleic acid extracts were also obtained from the University of Toronto, Canada and from the Oregon Health and Sciences University Brain Bank, Portland OR, USA.

### Statistics

In experiments *in vitro* we used cells: one PC12 line, one APPwt PC12 line and one APPsw PC12 line. In our manuscript “n” means the number of independent experiments (various days and various passages) *in vitro*. Statistical analysis of data derived from PC12 cells was performed with Graph Pad Prism version 5.0 (Graph Pad Software, San Diego, CA) using one-way analysis of variance (ANOVA) with Neuman-Keuls post-hoc test. Data are presented as means ± SEM. P<0.05 was considered significant.

The data from microarray analysis of mouse hippocampus was performed using a three-way ANOVA. The genes with an FDR <0.05 were considered statistically significant. P <0.05 was deemed as statistically significant. The “n” refers to the number of animals in the experimental group.

For statistical analysis of human DNA arrays, probe arrays were scanned with a confocal laser scanner (Agilent Technologies, Palo Alto, CA) at 570 nm, pixel intensities were measured, expression signals were analyzed and features extracted using a commercial software package (Microarray Suite ver. 5.0, Affymetrix). Data and statistical analyses were performed either at LC Sciences or at our own research facilities using MicroDB ver. 2.0, Data Mining Tool ver 3.0, Microarray Suite ver 5.0, LIMS (Affymetrix) and/or Genespring ver. 4.1.5 (Silicon Genetics, Redwood City CA, USA) bioinformatics algorithms. All resulting mRNA abundance data were collected and analyzed using Excel 2020 (Office 365) algorithms (Microsoft Corporation, Redmond WA, USA) [17,19,21]. Statistical significance was analyzed using a two‐way factorial analysis of variance (*p*, ANOVA; SAS Institute, Cary, NC). In these experiments the “n” refers to the number of post-mortem brain donors in the analyzed group.

## Results

To study the effect of elevated levels of Aβ peptides on cell cycle propagation in proliferating cells we used PC12 cell line which were stably transfected with: **(i)** an empty vector (PC12 line); **(ii)** the human wild-type *APP* gene (APPwt PC12 line); and **(iii)** the human *APP* gene congaing the Swedish mutation (APPsw PC12 line) [15]. As shown previously, compared to cells transfected with empty vector, the PC12 cells expressing human wild-type *APP* and mutant *APP* showed 2.8- and 4.8-fold higher production of Aβ peptides, respectively [22].

**Fig 1:**
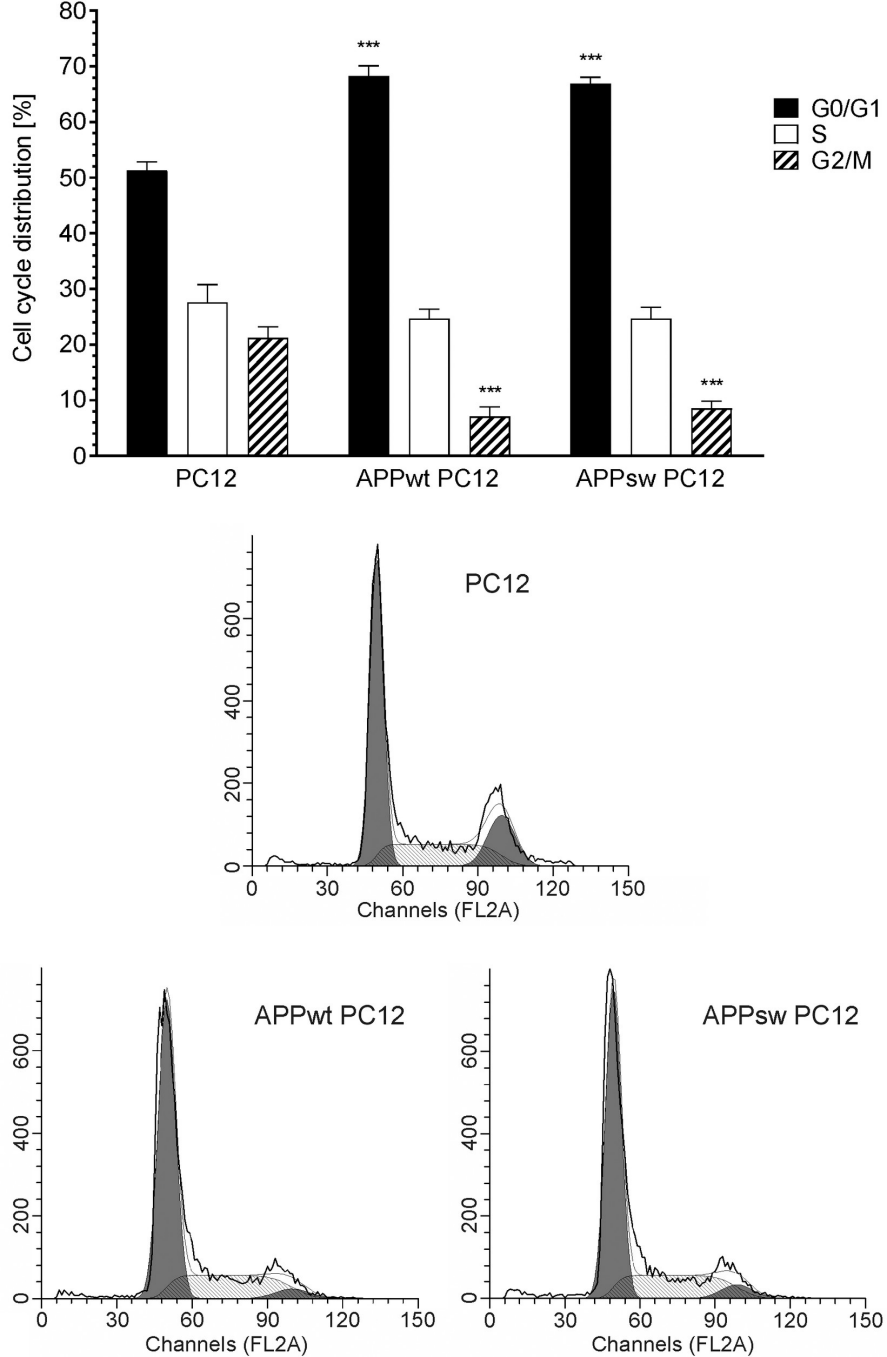
The effect of transfection with human *βAPP* gene on the distribution of the cell cycle. Flow-cytometric analysis for PC12, APPwt PC12 and APPsw PC12 cells. Representative histograms are presented. Data represent the mean value ± S.E.M. for 5 independent experiments (n = 5). *** p<0.001, compared to control PC12 cells.

As shown on Fig 1, in cell lines expressing human βAPP the cell cycle was disturbed. The fraction of cells in G0/G1 phase was increased, and the fraction of cells in G2/M phase was reduced, as compared to control cells. However, there was no difference between cell lines expressing human wild-type *APP* and human Swedish mutant *APP*.

**Fig 2:**
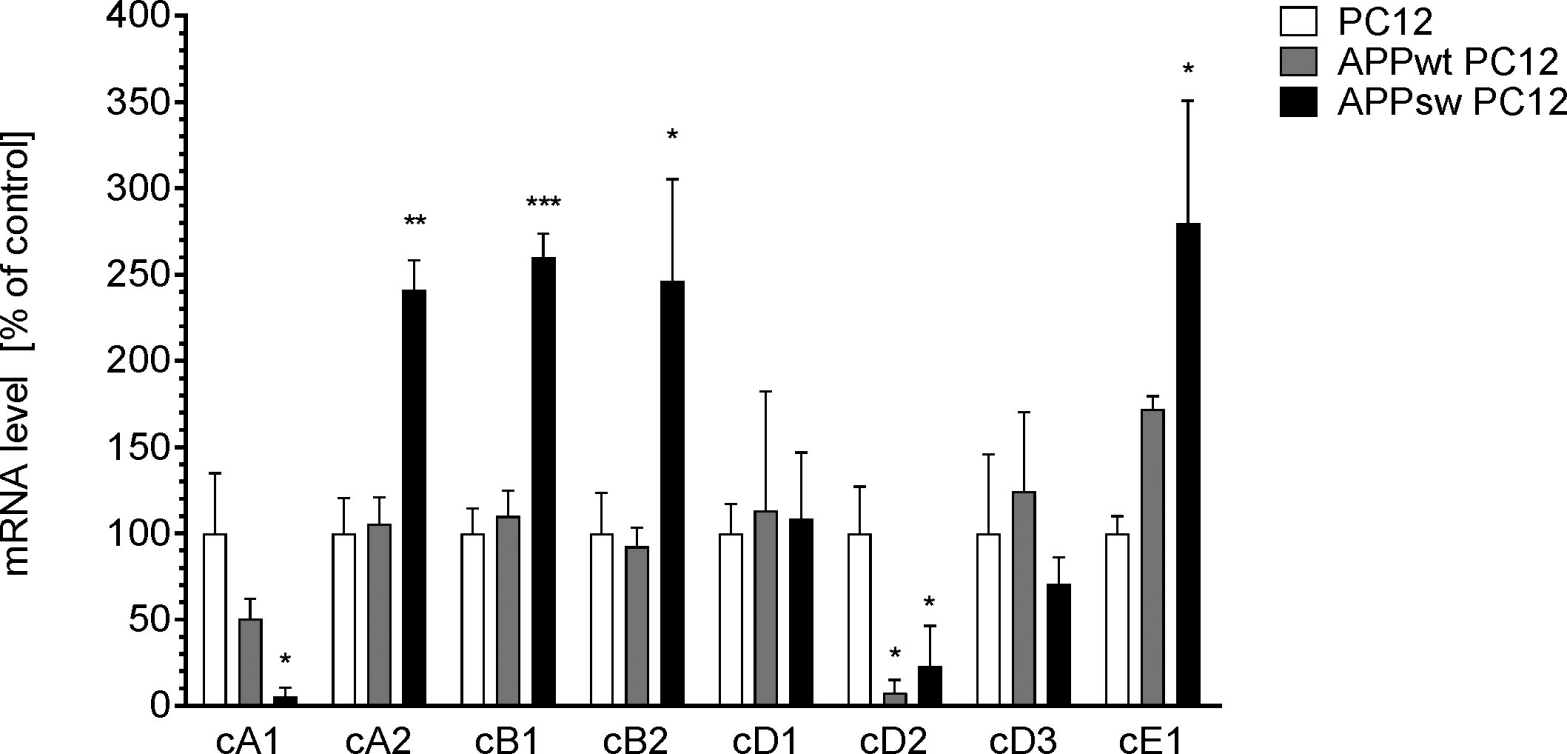
The effect of transfection with human *APP* gene on the level of mRNA for cyclin genes in PC12 cells. The level of mRNA for gene expression analysis in cell lines PC12, APPwt PC12 and APPsw PC12 using real-time PCR methods. Data represent the mean value ± S.E.M. for 3–4 independent experiments (n = 3–4). * p<0.05, **p<0.01, ***p<0.001 compared to control PC12 cells.

To reveal the mechanism of βAPP-evoked dysregulation of the cell cycle, the levels of mRNA for key genes involved in controlling cell-cycle, cyclins and cyclin-dependent kinases were quantified. As shown on Fig 2, in cells overexpressing wild-type *βAPP* only expression of cyclin D2 was significantly decreased, as compared to control cells. Overexpression of Swedish mutant *βAPP* also evoked a decrease in the expression of the gene for cyclin D2 and also for cyclin A1. Moreover, overexpression of Swedish mutant *APP* induced an increase in mRNA level for cyclins A2, B1, B2 and E1. Expression of *Cdk1*, *Cdk2*, *Cdk4*, *Cdk6*, *Cdk7*, *Cdk9*, and *Cdk10* was not significantly affected (S2 Table); we have demonstrated previously that expression of *Cdk5* was significantly increased in the APPsw cell line [23].

**Fig 3:**
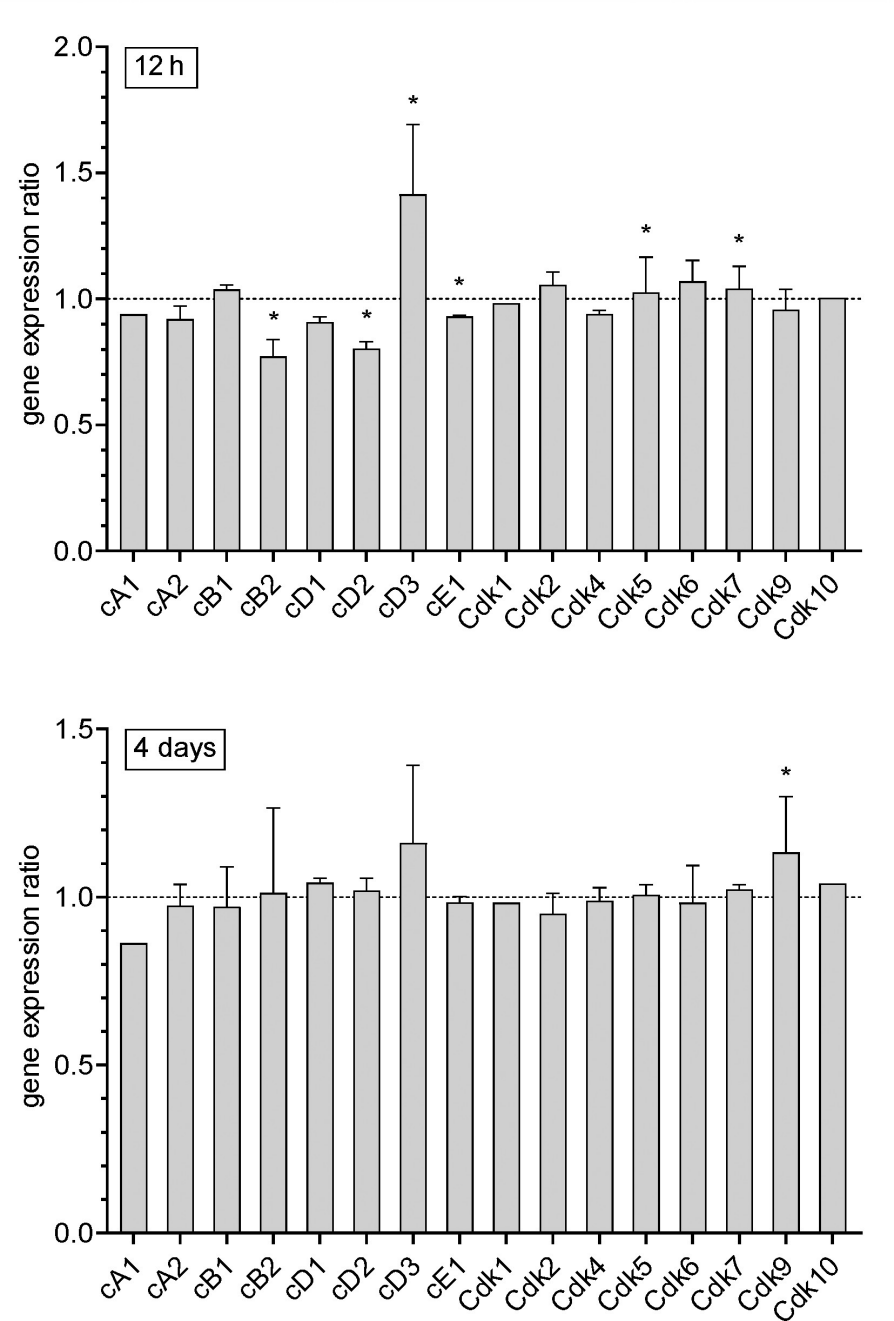
The effect of LPS-evoked systemic inflammatory response (SIR) on the level of mRNA for cyclins and Cdks in hippocampi of mice. Microarray analysis of expression of cell cycle-related genes in hippocampus 12 h and 4 days after peripheral administration of LPS. Gene expression ratio was calculated in comparison to control animals separately for each time point. n = 4; Data represent the mean value ± SD; *p<0.05, comparing to control group for at least one transcript.

Our previous studies demonstrated that several genes whose altered expression is involved in the pathomechanism of AD are also affected in the brain during the systemic inflammatory response (SIR) [16]. Therefore, to analyze if analogous changes in the expression of genes representing cell-cycle-regulating machinery occur during neuroinflammation, we applied the model of lipopolysaccharide (LPS)-induced systemic inflammation. We focused on the hippocampus, because this structure is affected both in AD and during systemic inflammation, as we have demonstrated in our previous studies [24–26]. A dose 1 mg/kg of LPS is commonly used in mouse models of systemic inflammation as it evokes a robust but transient response of the immune system [24–29]. All mice injected with LPS presented a transitory (up to 48 h post-injection) inflammation-associated illness. Moreover, as we demonstrated previously, 7 days after administration of LPS, some cognitive impairment was detectable by the new object recognition task [30]. The microarray analysis of gene expression pattern in mouse hippocampus was performed 12 h and 4 days after peripheral administration of LPS. As shown on Fig 3, the gene expression ratio for several cyclins and Cdks is changed. Twelve hours after administration of LPS, expression of genes encoding cyclin B2, D2, and E1 was decreased, whereas expression of gene for cyclin D3 was increased. Four days after injection of LPS expression of genes for cyclins was not changed. Among Cdks, expression of *Cdk5* and *Cdk7* was increased 12 h after LPS, and expression of *Cdk9* was increased 4 days after LPS. The expression of other Cdks analyzed was not changed (Fig 3).

**Fig 4:**
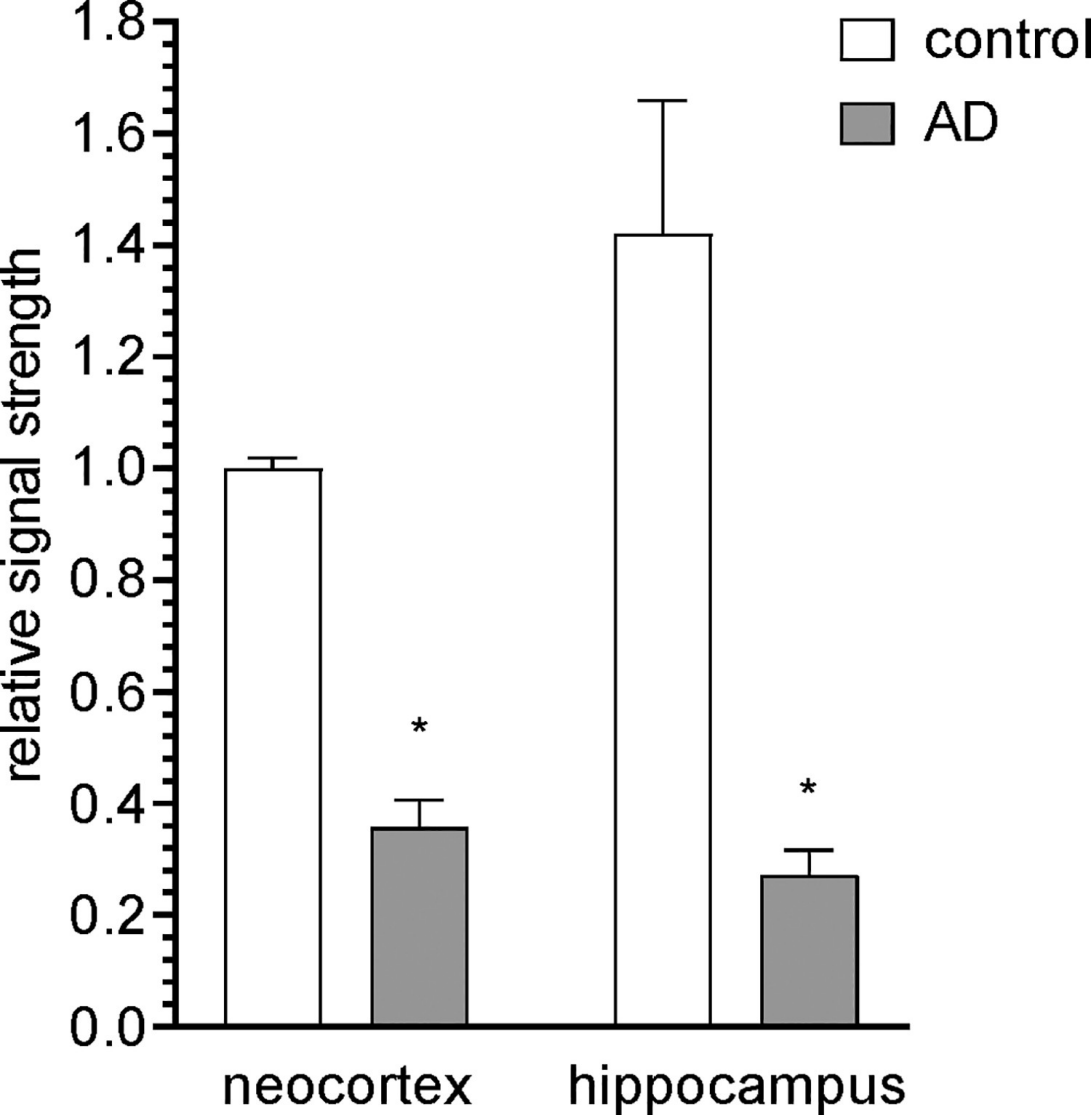
Cyclin D2 (cD2) mRNA levels in control and sporadic AD neocortex and hippocampus. Microarray-based analysis of cD2 levels in control (n = 4) and AD (n = 5) tissues based on unchanging β-actin control levels in the same sample; for ease of comparison of relative signal strengths the cD2 level in control human neocortex was set to 1.0; importantly there was no difference in age, gender, PMI or total RNA yield between the control and AD groups; (see section 2.7 under Materials and methods for details on AD and control tissues); no significant change in the abundance of cyclin B1, B2, D1, or D3 were noted in the human neocortex or hippocampus; the PMI is the death to brain tissue freezing interval at -81°C; wide bars represent the mean of control or AD samples and the error bars represent one standard deviation (SD) of that mean; * p<0.01 (ANOVA).

Cyclin D2 (cD2) was found to be abundantly expressed in control human brain neocortex and hippocampus but there was observed a statistically significant reduction of cD2 expression at the mRNA level in sporadic AD-affected human brain (Fig 4). The reduction of cD2 in AD was found to be down to 0.36-fold of control in neocortex and down to 0.19 of control in hippocampus. Interestingly, in control brain tissues the basal level of cD2 expression was 1.4-fold higher in the hippocampus when compared to age, gender and PMI-matched neocortex controls (Fig 4).

## Discussion

The major novelty of this study is demonstrating that the expression of cyclin D2 (cD2) is uniformly decreased in all tested conditions related to the pathomechanism of Alzheimer’s disease (AD), including Aβ toxicity, neuroinflammation and human post-mortem brain tissue from AD patients. The cD2 has been previously investigated in the brain development and neurogenesis, but it has been never analyzed in context of AD. These novel data opens the possibility on the potential role of cD2 in the pathomechanism of AD.

In our study we used three experimental models related to some aspects of pathomechanism of AD. APPwt PC12 cells produce medium amounts of Aβ peptides and may correspond to sporadic form of AD, and APPsw PC12 cells produce high amounts of Aβ peptides and may more closely correspond to genetic form of AD [31]. The cells reveal phenotype characteristic for AD, including hyperphosphorylation of tau, intracellular accumulation of aggregates of Aβ and release of Aβ to the culture medium [23,32]. Our next experimental model involving the systemic inflammatory response (SIR) activates in the brain neuroinflammatory processes and changes in gene expression patterns which resembles some changes observed in AD-affected brain [16].

The use of LPS is highly relevant in inducing neuroinflammatory signaling in experimental model of AD. Interestingly, gastrointestinal (GI) tract microbiome-derived LPS: **(i)** has been found within the cytoplasm of AD brain neocortex and hippocampus; **(ii)** has a progressively deleterious association with neocortical neurons in AD; **(iii)** restricts the expression of AD-relevant genes; and **(iv)** Aβ peptides appear to facilitate the entry of LPS specifically into the cytoplasm of neocortical neurons [33,34].

Our data demonstrate a significant downregulation of cD2 in experimental model of sporadic form of AD and in post-mortem sporadic AD neocortex and hippocampus. Changes in expression of cD2 and also other cyclins and cyclin-dependent kinases were also observed *in vitro* in a model of genetic forms of AD and in SIR. In a model of AD/Aβ toxicity we observed in APPsw PC12 cells a downregulation of not only cD2, but also cA1 and upregulation of some other cyclins as cA2, cB1, cB2, cE1 and also Cdk5 (as shown previously in [23]). However, in APPwt PC12 only gene expression encoding cD2 was found to be significantly decreased. Additionally, short term SIR transiently induced also downregulation of transcription of the gene for cD2 and concomitantly cB2, cE1, but activated gene expression for cD3 in the hippocampus 12 h after LPS injection, however 4 days after LPS only Cdk9 was altered. The common alteration in all investigated conditions (post-mortem human tissue and experimental models *in vitro* and *in vivo*) is the significant downregulation of cD2.

Up until now no data exist on the role and expression of cD2 in brain aging and in sporadic AD brain. The current study demonstrated that cD2 expression is reduced in both AD-affected neocortex and hippocampus when compared to age-, gender and PMI-matched controls. The previous study by Kowalczyk et al. showed that cD2 is the only one D-type cyclin expressed in neuronal precursors in adult hippocampus [35]. The study demonstrated that cD2 mutations can completely eliminate neurogenesis in the adult brain. Adult neurogenesis plays a significant role in neurodegeneration by formation of new neurons in dentate gyrus of the hippocampus and in the olfactory bulb during life span /aging and, as indicated several other studies, after different types of injury, including brain trauma [36,37]. The lack of cD2, which was experimentally evoked by gene ablation, leads to absence of proliferation of neuronal precursor cells exclusively in adult brain in opposition to developmental brain where cD2 function could be replaced by other cyclins D (cD1 or cD3) [35,38]. On the other hand, mutant mice expressing only cD2, but not cD1 and cD3, presented abnormal neurological reflexes leading to death by the end of the first day of life [39]. The further studies have suggested the evolutionary importance of cD2 in larger mammals where expansive intermediate progenitor divisions enable the generation of larger cerebral cortices [40].

Besides regulating and controlling the cell cycle, the cD2 may play an important role in regulation of Cdk5/p35 activity [41]. Cdk5 plays an important role in development of the central nervous system (CNS), in cellular differentiation, in synaptic function, and in memory [42]. Cdk5 is overexpressed in post-mitotic neurons and is not involved in direct regulation of cell cycle but it seems to play an important role in pathomechanism of AD which is suggested also on the basis of our previous studies [23,26,43]. In pathological conditions Cdk5 is over-activated and could be responsible for excessive phosphorylation of βAPP, tau protein, neurofilament proteins, mitochondrial dysfunction, synaptic degeneration and neuronal death. Inhibition of Cdk5 using RNA interference protects against impairment of memory and neuronal loss in progressive and terminal neurodegenerative conditions [44].

Numerous investigations demonstrated the alteration in expression of several cyclins, Cdks and the effects of several inhibitors of Cdks in experimental models of AD and in AD brain, but up till now no information exists on cD2 and cD1, cD3 in AD [45–50]. Previous studies have described the reactivation of some cell cycle processes in post-mitotic neurons which may facilitate neurofibrillary degeneration leading to formation of neurofibrillary tangles (NFT) in AD brain and in mitotic cells in culture [47,49]. Aberrant re-expression of Cdk1/cB1 in NFT-bearing neurons has been suggested to be responsible for the generation of M-phase phospho-epitopes in NFT in AD brain [47]. The expression of Cdk4 and cyclin D was found previously in the brains of AD patients [48,50]. Several previous reports have indicated that alterations of two pathways involving cyclin dependent kinases may be responsible for neuronal degeneration and death in AD. Monaco and Vallano suggested that the crucial pathway which is connected with aberrant reactivation of cell cycle is not correlated with changes of neuronal function [51]. Several other studies proposed that alteration of cyclins and Cdks could be responsible for neurodegeneration and AD pathology. It has been found in dividing non-neuronal cells that the translocation of MAPK to the nucleus can stimulate the Cdk4/cD complex which is responsible for the transition of cells from G0 to G1 phase of cell cycle [52]. The question arises, whether lower expression of cD2 observed in human AD brain could be responsible for the alteration of cell cycle and for neurodegeneration or brain cell death. Another important question is, if the lower expression of cD2 in human AD brain may decrease neurogenesis and affect neuroprotective processes concomitantly activated with neurodegeneration. It is now well known that in AD brain several mitogenic pathways including those regulated by p21ras/MAP kinase are activated, leading to hyperphosphorylation of tau protein and for changes in βAPP metabolism and excessive Aβ liberation [52]. Herein was described a significant elevation of cyclin C in neurons and astrocytes in AD and also an increased level of interacting protein Cdk8 exclusively in astrocytes. On the basis of numerous data obtained in dividing non-neuronal cells it is known that the cell cycle mechanism is tightly regulated and that cyclin/cyclin kinases complexes play a crucial role in homeostatic cell function. Our data suggest that excessive Aβ peptide liberation and accumulation in brain cells could be responsible for the observed downregulation of cD2 in AD brain. Using PC12 cells transfected with human wild-type *APP* (which may correspond to sporadic form of AD) we have observed lower expression of cD2 and this was only one alteration found in the transcription of genes related to investigated cell cycle proteins. However, in APPsw PC12 cells which accumulate and release higher amounts of Aβ peptide, compared to the APPwt PC12 line, not only the lower expression of cD2 but also cA1 and concomitantly higher gene expression for cA2, cB1 cB2 and cE1 was observed. Our previous study demonstrated the upregulation of Cdk5 in APPsw PC12 [53]. Data on Aβ peptide and/or Aβ peptide fragment effects on cell cycle changes over time are also relatively sparse. Neurotoxic fragments of the full Aβ peptide Aβ_25–35_ have been shown to enhance the number of neuroblastoma cells (SH-SY5Y) in the S phase and to decrease the population of cells in the G_2_/M phase [54]. In another recent study, Tsai et al. reported that Aβ_25–35_ prevented cell-cycle re-entry in human neuroblastoma SH-SY5Y cell line, and increased level of cyclin D1 [55]. However, the authors studied only the short-term effect (24 h) of exogenous Aβ (short fragment 25–35) on cells, whereas in our study the long-term exposition to endogenous Aβ (intra- and extracellular) was analyzed [32,56]. Therefore, the differences in the experimental approach may be the reason of discrepancy in cD1 expression. The dynamics of gene expression is time-dependent, and alterations observed after 24 h should be considered as transient. Aβ_1–42_ oligomers have also been shown to evoke re-activation of the cell-cycle in cortical neurons in primary culture and this activation was dependent on AKT and mTOR signaling pathway [57]. It was also recently demonstrated that specific Aβ peptide species /oligomers affect the cell cycle and growth of human cancer cells [58]. Other studies indicated that pro-inflammatory processes in the brain may also activate cell-cycle [36,37]. The studies on traumatic brain injury (TBI)-evoked neuroinflammation indicate cell-cycle activation (CCA) which may lead to death of neurons and oligodendroglia, and to activation of microglia and astrocytes which subsequently may be responsible for increased neuroinflammatory signalling. The Cdk5 inhibitor roscovitine and the novel inhibitor of CDKs CR8 have been shown to significantly decrease microglial activation, astrocytosis and neuronal loss after TBI. On the basis of these data authors suggested that alterations in CCA may be involved in neurodegeneration and may be responsible in part for AD-relevant neurological dysfunction [36,37].

Another important issue is the correlation of cell cycle-related changes and tau phosphorylation. For many years tau pathology/toxicity was underestimated and considered as secondary event comparing to the neurotoxicity of Aβ peptides, but many recent studies suggest a “dual pathway” or “dual cascade” hypothesis highlighting the role of factors upstream of both Aβ peptides and tau [59,60]. In our previous study we demonstrated that compared to control PC12 lines, tau phosphorylation at Ser396 is increased exclusively in APPsw PC12 line, but not in APPwt PC12 lines [23]. Also in the SIR model, tau phosphorylation at Ser396 is increased [26].

In summary, our results provide evidence for the first time that the expression of cyclin D2 is reduced in *in vitro* experimental cellular models of the sporadic/genetic form of AD, in murine model of LPS-induced systemic inflammatory response (SIR), and in multiple post-mortem samples of sporadic AD brain compared to age- and gender-matched controls. Moreover, our data indicate that both toxicity of Aβ peptides and neuroinflammatory signalling may be responsible for the observed lower expression of cD2 in anatomical regions of the brain targeted by the neuropathology of AD. The question arises as to what kind of specific or ancillary roles cyclin D2 could play in the pathomechanism of AD, and what cD2-targeted treatments and/or therapies could be the most efficacious in the clinical management of AD and perhaps other progressive neurodegenerative disorders.

## Supporting information

S1 Table The list of primers’ sequences used in the study. (DOCX)

S2 Table The effect of βAPP transfection on the level of mRNA for cyclin-dependent kinases genes. The level of mRNA for studied genes in cell lines PC12, APPwt PC12 and APPsw PC12 was analyzed using real-time PCR methods. Data represent the mean value ± S.E.M. for 3**–**4 independent experiments. * p<0.05, comparing to control PC12 cells which were presented as 100%.
(DOCX)
