# Supplementary material for: Down-regulation of cyclin D2 in amyloid β toxicity, inflammation, and Alzheimer’s disease
Source: PLoS One. 2021 Nov 18;16(11):e0259740. doi: 10.1371/journal.pone.0259740 (PMC8601534; doi:10.1371/journal.pone.0259740)
Supplement: S1 Table — (DOCX) [file pone.0259740.s001.docx]

**S1 Table. The list of primers’ sequences used in the study.**

gene forward primer reverse primer

Cyclin A1 (*Ccna1*) 5’-CGCAACGATCAGATGTTTTTCT-3’ 5’-CCCGTTGTCAGGGAGTACTTTC-3’

Cyclin A2 (*Ccna2*) 5’-TGCACCAACAGTAAATCAGTTCCTTA-3’ 5’-TTAACTCTCCCAAAAACATCGCTAA-3’

Cyclin B1 (*Ccnb1*) 5’-ACGGTGAATGGACACCAACTC-3’ 5’-CTGCATCACAGGAAGCAGAGAT-3’

Cyclin B2 (*Ccnb2*) 5’-TCCAGCCCACCAAAGCAA-3’ 5’-CTGCACTGGTTTCACAGAAGCT-3’

Cyclin D1 (*Ccnd1*) 5’-GCCAGAGGCGGATGAGAAC-3’ 5’-GGCACAGAGGGCCACAAA-3’

Cyclin D2 (*Ccnd2*) 5’-CGCTCTGTGTGCTACCGACTT-3’ 5’-CGCTTCCAGTTGCGATCA-3’

Cyclin D3 (*Ccnd3*) 5’-CTGTGATTGCGCACGACTTC-3’ 5’-CCGGTCACTGGGCAGAGA-3’

Cyclin E1 (*Ccne1*) 5’-AGACTTACCTGAGAGATGAGCACTTTC-3’ 5’-CATCAGCCAGTCCAGAAGAACTG-3’

Cyclin-Dependent Kinase 1 (*Cdk1*) 5’-CGGTCGCCAGAGGTGTTG-3’ 5’-GCTCTGCAAATATGGTCCCTATG-3’

Cyclin-Dependent Kinase 2 (*Cdk2*) 5’-CGGACGGAGCTTGTTTATCTCA-3’ 5’-GGGCTGCTTTGGCTGGAAAT-3’

Cyclin-Dependent Kinase 4 (*Cdk4*) 5’-CGGACATACCTGGACAAAGCA-3’ 5’-ACTGGCGCATCAGATCCTTAA-3’

Cyclin-Dependent Kinase 6 (*Cdk6*) 5’-TCTTCCCAGACAGGCTTTTCA-3’ 5’-TCGTCGATGTCTGTCA-3’

Cyclin-Dependent Kinase 7 (*Cdk7*) 5’-AATCGTCCAGGGCCAACAC-3’ 5’-GGCTGGATTTCACTGTTCCTTT-3’

Cyclin-Dependent Kinase 9 (*Cdk9*) 5’-GTGCATCATGGCAGAGATGTG-3’ 5’-GCTGGTGCTGCTCTGTGTTG-3’

Cyclin-Dependent Kinase 10 (*Cdk10*) 5’-GCTTCGAGGCCTTCAGTACCT-3’ 5’-TCATGAGCAAGTTGGAACACCTT-3’

Primers were designed using Primer Express 3.0 from Applied Biosystems.
