# Supplementary material for: Down-regulation of cyclin D2 in amyloid β toxicity, inflammation, and Alzheimer’s disease
Source: PLoS One. 2021 Nov 18;16(11):e0259740. doi: 10.1371/journal.pone.0259740 (PMC8601534; doi:10.1371/journal.pone.0259740)
Supplement: S2 Table — The level of mRNA for studied genes in cell lines PC12, APPwt PC12 and APPsw PC12 was analyzed using real-time PCR methods. Data represent the mean value ± S.E.M. for 3–4 independent experiments. * p<0.05, comparing to control PC12 cells which were presented as 100%. (DOCX) [file pone.0259740.s002.docx]

**S2 Table. The effect of βAPP transfection on the level of mRNA for cyclin-dependent kinases genes.**

|  | PC12 | | APPwt PC12 | | APPsw PC12 | |
| --- | --- | --- | --- | --- | --- | --- |
| Cdk1 | 100.00 | ± 8.27 | 89.05 | ± 5.78 | 74.80 | ± 10.89 |
| Cdk2 | 100.00 | ± 6.10 | 84.70 | ± 10.10 | 75.27 | ± 4.08 |
| Cdk4 | 100.00 | ± 5.16 | 91.49 | ± 6.18 | 82.99 | ± 1.03 |
| Cdk6 | 100.00 | ± 7.75 | 94.32 | ± 12.00 | 84.09 | ± 3.94 |
| Cdk7 | 100.00 | ± 14.05 | 98.88 | ± 13.47 | 84.40 | ± 35.74 |
| Cdk9 | 100.00 | ± 4.58 | 97.29 | ± 9.98 | 88.54 | ± 11.27 |
| Cdk10 | 100.00 | ± 5.03 | 103.65 | ± 6.08 | 113.63 | ± 11.87 |

The level of mRNA for studied genes in cell lines PC12, APPwt PC12 and APPsw PC12 was analyzed using real-time PCR methods. Data represent the mean value ± S.E.M. for 3-4 independent experiments. * p<0.05, comparing to control PC12 cells which were presented as 100%.
